# Physicochemical and Sensory Properties of Vegan Gummy Candies Enriched with High-Fiber Jaban Watermelon Exocarp Powder

**DOI:** 10.3390/foods12071478

**Published:** 2023-03-31

**Authors:** Mohammad Tarahi, Malihe Mohamadzade Fakhr-davood, Shiva Ghaedrahmati, Sahar Roshanak, Fakhri Shahidi

**Affiliations:** Department of Food Science and Technology, Faculty of Agriculture, Ferdowsi University of Mashhad, Mashhad 9177948978, Iran

**Keywords:** gummy candy, functional food, high-fiber product, fruit byproduct, confectionery industry

## Abstract

The customer demand for healthier, fortified, and vegan products has recently received much attention. In this study, the vitamin C content, total phenolic compounds, total flavonoid, and anthocyanin, as well as the antioxidant activity of Jaban watermelon exocarp (JWE) powder were first investigated. Then, the gummy candies (GCs) were prepared with different concentrations of JWE powder (20, 35, and 50%), citric acid (0.75 and 1%), and agar gum (0.5 and 1%), and their physicochemical and sensory properties were evaluated. The results showed that the moisture content and water activity of GCs decreased, while the pH value increased with the increasing concentration of JWE powder. Moreover, the GCs became brighter and more yellowish. The rheological and textural analysis indicated that the viscosity and hardness of GCs improved in higher JWE powder levels (0.457 to 1.550 Pa·s and 1667 to 7232 g, respectively). Furthermore, the highest panelists’ score was given to the GC 5 sample with 35% JWE powder, 0.75% acetic acid, and 0.5% agar gum.

## 1. Introduction

The confectionery industry is one of the most important food sectors worldwide, which has approximately USD 82.3 billion market value with a growth rate of 3.6% annually, according to 2018 data. In addition, the candy industry is a third major part of the confectionery industry, with a high dynamic market on a global scale [1,2]. Gummy candies (GCs) are foodstuffs whose main ingredient is gelling agents (gelatin, starch, gums, and pectin), sweetening agents (sucrose, glucose, corn syrups), acids, aromas, and food pigments [1]. The consumer has recently expanded toward healthy, fortified, functional, sustainable, and vegan-oriented products. Therefore, the consumers prefer healthier GCs with lower sugar content, vegan-friendly components, and the addition of dietary fiber, vitamins, antioxidants, phenolic components, probiotics microorganisms, etc. [2,3]. To meet such demands, some strategies are used to improve GC formulations and create specific functional properties by using sugar alternatives [4], natural flavors and colors [5,6], bioactive compounds [1], incorporation of dietary fiber [7,8], probiotic microorganisms [4], and substitutes for gelatin [2,3].

Veganism and different plant-based diets have been a top trend in the last few years [9]. Some studies suggest that livestock industries can be the reason for some environmental and health issues, as well as ecological problems, such as greenhouse gas emissions, land degradation, surface and groundwater pollution, and the salinization of soils. Moreover, a meat- and dairy-based diet is naturally higher in fat and cholesterol. Therefore, too much production and utilization of meat and dairy have a significant impact on the environment, human health, and the economy [10,11]. On the other hand, some evidence claimed that vegetarianism is linked to improving nutrition and health conditions. Both vegan and vegetarian diets may lower body mass index (BMI), risks of certain cancers, LDL-cholesterol, glucose levels, and blood pressure, as well as reduce the risk of diabetes and cardiovascular diseases [12,13]. Traditional GCs commonly used gelatin as a gelling agent, a derivative of collagen from varied sources such as pigskin and bovine hides. Although gelatin provides diverse functional characteristics such as firming, consistency, and gel-forming, it is becoming unacceptable due to the high demand for vegan, halal, and kosher-certified food products. In this regard, some nonanimal hydrocolloids and gelatin replacers such as pectin, agar, starch, carrageenan, and Arabic gum were also successfully used in some studies for candies and GC products [2].

Recently, the application of dietary fiber has grown to enhance the nutritional value of food products [14]. The most popular dietary fiber products are those derived from cereals. However, the consumption of high dietary fiber fruits such as citrus, apples, melons, and others has increased over the past decade. Fruit dietary fiber concentrates have higher nutritional value, bioactive compounds, and fiber content than cereal derivatives [15]. In addition, byproduct recovery of food and agriculture waste has become a growing challenge because of the increasing interest in producing new functional foods with higher levels of dietary fiber, bioactive compounds, polyphenols, and natural antioxidants, as well as the attention to environmental sustainability [16]. Moreover, one of the best advantages is that they can be ready for use after only minimal processing such as drying. For this reason, the studies on the usage of fruit wastes in confectionery products have increased in recent years [14].

Some types of fruits are consumed due to their taste and nutritional value, such as watermelon. In addition, more than 40% of the fruit’s total mass, including the rind, pulp, and seeds, is wasted and not edible, which can be used in food manufacturing as a cheap, high nutritional value, and eco-friendly component [17]. Watermelon (*Citrullus lanatus*) has great economic importance with the production of approximately 93.7 thousand million tons a year worldwide [18]. The Jaban watermelon originally belongs to a village called “Jaban” near Damavand, Iran, and it is produced for nuts and the rest remains as waste. Our previous study showed that the Jaban watermelon exocarp (JWE) can be a good source of dietary fiber (15.52%) and minerals (especially, Fe, Al, Zn, and Cu), which introduces it as a new functional ingredient for food industry applications [19].

Some studies showed the possible usage of various fruit skins and peels such as grape, pineapple, and papaya for the production of GCs [20]. However, to the best of our knowledge, there is no investigation about the usage of the watermelon rind and exocarp in GC processing. Therefore, this research aimed to develop novel functional GCs with different percentages of the JWE powders (20, 35, and 50%), citric acid (0.75 and 1%), and agar gum (0.5 and 1%), as well as evaluate their physicochemical and sensory properties.

## 2. Materials and Methods

### 2.1. Materials

The Jaban watermelon was purchased from a local market in Neyshabur, Iran. All chemicals and reagents were purchased from the Merck (Darmstadt, Germany) and Sigma Aldrich (St. Louis, MO, USA) companies. All materials used in this study were of analytical grade.

### 2.2. JWE Powder Preparation

The JWE powder was prepared according to the method described by Ho et al. [21]. Briefly, the white watermelon rinds were washed and cut into equal pieces and then dried for 24 h at 50 °C. The dried samples were milled and sieved through a #25 mesh and kept in the refrigerator (4 °C).

### 2.3. JWE Powder Characterization

#### 2.3.1. Vitamin C Content

Ascorbic acid (AA) was evaluated by the 2,6-dichloroindophenol titrimetric method according to AOAC method No. 967.21 [22].

#### 2.3.2. Total Phenolic Compounds Content

The total phenolic content (TPC) of JWE powder was measured by Folin–Ciocalteu assay based on the method described by Pinelo et al. [23]. Briefly, 50 mL of 80% methanol was added to 2.5 g of JWE powder and stirred at 240 rpm for 24 h and filtered. Then, 500 µL of diluted extraction, 2.5 mL sodium carbonate 7.5%, and 2.5 mL Folin–Ciocalteu reagent were mixed. The mixture was heated for 15 min at 45 °C, and the absorbance was measured at 765 nm, using a UV/vis Spectrophotometer (IRMECO, U2020, Geesthacht, Germany). The TPC was determined via a calibration curve prepared with a series of gallic acid standards. Results were expressed as mg of the gallic acid equivalent per g of JWE powder weight.

#### 2.3.3. Total Flavonoid

The procedure described by Roshanak et al. [24] was pursued to determine the total flavonoid content (TFC) of JWE powder based on the formation of a flavonoid–aluminum complex. A volume of 0.5 mL of extract solution and 2 mL of distilled water were mixed with 0.15 mL of 5% NaNO_2_. After 6 min, 0.15 mL of 10% AlCl_3_ was added to the mixture and allowed to stand for 6 min. In addition, 2 mL of 4% NaOH was added to the mixture. The final volume of the mixture was adjusted to 5 mL with distilled water. The mixture was allowed to stand for 15 min, and the absorbance was measured at 515 nm. TFC were calculated based on the standard curves, and the final values were expressed as mg of the catechin equivalent per 100 g of JWE powder weight [25].

#### 2.3.4. DPPH Radical-Scavenging Activity

The total antioxidant activity (TAA) of JWE powder was measured by 2,2-diphenyl-1-picrylhydrazyl (DPPH) free radicals according to the method described by Roshanak et al. [24]. Briefly, 0.2 mL of extract solution was mixed with 0.8 mL of 80% methanol to prepare the stock solution ranged from 5 to 0.5 mg/mL. A volume of100 µL of diluted solutions and 5 mL of 0.1 mM DPPH solution were mixed, and the volume of the solution was adjusted to 10 mL with 80% methanol. The solution was incubated for 1 h in a dark place at room temperature, and the reduction in DPPH was recorded at the absorbance of 517 nm. The control samples contained all the reagents without sample extract. The DPPH inhibition was calculated using Equation (1):

DPPH inhibition activity (%) = [(A control − A sample)/A control] × 100(1)

Butyl hydroxy anisole (BHA) was used as a standard reference [26].

#### 2.3.5. Total Anthocyanin Content

The total anthocyanin content (TAC) of JWE powder was determined by the method of Yang et al. [27] and Camelo-Méndez et al. [28] based on the pH-differential method. Two dilutions were prepared, one in 0.025 M KCl buffer, pH 1.0; and another in sodium acetate buffer 0.4 M, pH 4.5. Then, the mixtures were balance in the dark for 1 h. The absorbance of each mixture was measured at 520 nm and 700 nm, respectively, and calculated by Equation (2):

A = (A_520_ − A_700_)_pH 1.0_ − (A_520_ − A_700_)_pH 4.5_ (2)

TAC of JWE sample was determined using Equation (3):

TAC (mg cyanidin-3-glucoside/100 g) = (A × MW × DF × V)/(ε × L × Wt × 100)(3)

where MW is cyanidin-3-glucoside weight (449.2), DF is dilution factor, V is final volume (mL), ε is cyanidin-3-glucoside molar absorptivity (26,900/cm·mol), L is the cell (cuvette) length (usually 1 cm), and Wt is extract weight (g).

### 2.4. GCs Production

The high-fiber GC samples were prepared with different levels of JWE powder (20, 35, and 50%), citric acid (0.75 and 1%), and agar (0.5 and 1%). In addition, the amounts of other components were obtained by pretesting and trial and error, as shown in Table 1. For this purpose, JWE powder was first mixed with water, then citric acid and sugar were added to the mixture. After that, the mixture of Arabic gum and starch was added and mixed well. The obtained mixture was placed in a water bath to reach a temperature of 80 °C. Then, the agar gum was added to the mixture. After mixing well, the mixture was poured into the molds. After the mixture reached room temperature, it was refrigerated for 4 h at 4 °C. Finally, the GCs were removed from the molds and placed in a dryer (Soroush Medical Co., Esfahan, Iran) at 25 °C for 24 h.

### 2.5. GCs Characterization

#### 2.5.1. Determination of Moisture Content and Water Activity

The moisture content of GCs was determined after dehydration of 1 g sample to a constant weight using the oven at 60 °C (Memmert UN30, Schwabach, Germany). The water activity of samples was measured at 25 °C using a water activity meter (Lab Master-aw, Novasina, Switzerland).

#### 2.5.2. Determination of pH Value

The pH value was measured by dissolving 1 g of sample in 10 mL distilled water (50 °C) using a digital pH meter (Metrohm 913, Herisau, Switzerland).

#### 2.5.3. Analysis of Color

The color of GCs was indicated by image analysis, referring to color factors; L* (lightness/brightness), a* (redness/greenness), b* (yellowness/blueness). The samples were put in a box (50 × 50 × 100 cm) which was prepared with eight fluorescent lamps with a radiation angle of 45° to each sample. The samples were photographed using a digital camera (Canon, EOS 1000D) with a distance of 20 cm and then analyzed by ImageJ (1.52v, NIH, Bethesda, MD, USA) software.

#### 2.5.4. Viscosity Properties

The apparent viscosity was determined using a rotational viscometer (Bohlin Model Visco 88, Bohlin Instruments, Cirencester, UK) equipped with C14 measuring spindles, as described by Sanchez et al. [29] with some modifications. The samples were transferred to the sample compartment, and the instrument was programmed to set the temperature at 70 °C and equilibrate at 117/s for about 2 min in the shear rate value from 14 to 150 s^−1^. Silicone oil was applied to the surfaces of samples to avoid evaporation.

#### 2.5.5. Textural Properties

The texture properties of GCs were performed using a TA-XT2i Texture Analyzer (Stable Micro Systems, Surrey, England) as described by Amjadi et al. [30] with some modifications. Each sample was compressed twice (30% of its original height) with a TA3/1000 flat cylindrical probe (38 mm in diameter), at a speed of 2 mm/s, trigger force of 10 g, and the delay between two compressions was 5 s. The obtained reports for this test include hardness, springiness, adhesiveness, chewiness, cohesiveness, and gumminess.

#### 2.5.6. Sensory Acceptability

Sensory evaluations of the GCs (color, texture, chewiness, flavor, aroma, and overall acceptability) were performed using ten (equal number of male and female) semitrained panelists in the range of 18–25 years old, who were assistants in the Department of Food Science and Technology, Faculty of Agriculture, Ferdowsi University of Mashhad, Iran. Individual characteristics of the GCs were evaluated by five-point hedonic scale (1 = absolutely not acceptable; 2 = not acceptable; 3 = acceptable; 4 = desired; 5 = highly desired). The study was reviewed and approved by the Ferdowsi University of Mashhad IRB and informed consent was obtained from each subject prior to their participation in the study.

### 2.6. Statistical Analysis

A completely randomized factorial design with three replicates was conducted for this research. Data were analyzed by Minitab 16 Statistical Software (Minitab Inc., State College, PA, USA). One-way analysis of variance (ANOVA) was performed using a Tukey’s test at a 5% level of significance to compare the sample means.

## 3. Results and Discussion

### 3.1. Chemical Composition and Antioxidant Activity of JWE Powder

Phenolic compounds have shown a wide range of cumulative biological effects, including anti-inflammatory, antibacterial, vasodilator actions, anticarcinogenic, antiviral, antithrombotic, antiallergic, and hepatoprotective [31]. The chemical, biochemical, clinical, and epidemiological evidences support the chemo-protective effects of phenolic substances against oxidative stress facilitated disorders [32]. In recent decades, flavonoids have been the focus of much research due to their potential as health-promoting phytochemicals. Flavonoids exhibit antioxidant and antimicrobial properties and have been investigated extensively regarding their ability to lower the risk of cardiovascular diseases [33]. The antioxidant system is one of the most important defense mechanisms of saliva against free radicals. One of the most important antioxidants is vitamin C, which effectively treats and prevents oxidative stress [34]. Watermelon rind (exocarp) powder contains a substantial level of phenolic antioxidants [32]. The vitamin C content was the main antioxidant compound found in JWE powder, with a value of 55.19 ± 0.41 mg/100 g (Table 2). The recommended dietary allowance (RDA) for vitamin C ranges from 15 to 75 mg for children, 75 mg for adult women, 90 mg for adult men, and 85–120 mg for women who are pregnant or breastfeeding [35]. The TFC in JWE powder (36.21 ± 0.12 mg catechin/100 g) was lower than the TPC, which was 52.62 ± 0.34 mg gallic acid/g. Moreover, the TAA of JWE powder was 25.06 ± 0.27%, which was determined by DPPH assay. In addition, the TAC in JWE powder was 2.56 ± 0.03 mg cyanidin-3-glucoside/100 g (Table 2). Previously, Al-Sayed and Ahmed [36] showed the TAA of 39.7 and 12.53% for watermelon rinds and sharlyn melon peels, respectively, which is aligns with our results. In addition, Abdulazeez et al. [37] demonstrated the TFC of 173.78 to 174.56 mg/100 g and TAA of 35.24 to 38.73% for different watermelon rinds. However, Kistriyani et al. [38] reported that the TAC, TFC, and TPC of red watermelon rind were 0.0334, 0.7369, and 0.3669 mg L^−1^, respectively. The different results may be due to the various botanical sources, production and storage conditions, and measurement methods.

### 3.2. Physicochemical Properties of GCs

#### 3.2.1. Moisture Content and Water Activity

Water is an essential ingredient in food products, which is closely related to the shelf life and the quality of the products. The moisture content of the foodstuffs is an important factor that can affect microbial spoilage and also the physical appearance [39]. The moisture contents of GCs were in the range of 23.3 to 29.4 g/100 g (Table 3). The results showed that the addition of JWE powder and citric acid can significantly decrease the moisture content of the GCs (*p* < 0.05), which was more pronounced at higher concentrations of JWE powder. However, the agar gum did not significantly change the moisture content of GCs.

Water activity is another important parameter for confectionery products due to its effect on the shelf life, quality, and sensory attributes of the products [40]. Although the previous studies represented the water activity of confectionery products at a range of 0.54–0.66, the GC products usually have higher water activity because of their soft and jelly nature, which ranges from 0.6 to 0.9 [40,41]. According to Table 3, the water activity of the GCs decreased from 0.81 to 0.69 with the addition of JWE powder.

Previously, Cappa et al. [8] showed that the moisture content and water activity of fruit candies were reduced by adding the grape skin powder. In addition, Ali et al. [7] reported that the strawberry and red beetroot fibers could reduce the moisture content of jelly candies, which is consistent with our findings. These results indicate that the presence of JWE powder in GCs and its interaction with the other components can reduce the percentage of moisture in the GCs. In other words, the samples with higher amounts of JWE powder had less free water due to the presence of more fiber, which can retard the growth of microorganisms and enhance the final product’s shelf life [40]. Moreover, the interactions of citric acid and the gelatinized starch during the thermal process could influence the moisture content and water activity of GCs [42].

#### 3.2.2. pH Value

Physical parameters such as pH are often determined to control the degree of hydration in confectionery factories. In addition, it is an important factor in preventing microorganisms’ growth, and GCs are usually classified as acidic food products [41,43]. As shown in Table 3, the pH values of GCs were generally increased by the addition of the JWE powder and citric acid (*p* < 0.05). The lowest pH value was related to the GC 4 sample (2.370), and the highest was related to the GC 9 sample (3.205). Romo-Zamarrón et al. [20] reported that adding the pineapple peel powder to the GC formula decreased the pH of the final product. However, the pH values of GCs increased with the addition of the papaya peel powder. These phenomena may be related to the higher acidity value of pineapple than papaya. Moreover, Ali et al. [7] represented that the jelly candies enriched with strawberry and red beetroot fibers had lower pH values than the control sample, which could be due to the citric acid present in fruits.

#### 3.2.3. Color

The optical properties of confectionery products have a significant effect on their overall perception and consumption. Moreover, colorants have a crucial effect on the aroma, gel texture, and storage stability of the final product [1]. In this study, the effects of different treatments on the color parameters (L*, a*, and b*) of GCs were investigated and shown in Table 4. The L* value indicates the brightness of samples, which varies from black to white, the a* value indicates color changes from red (positive values) to green (negative values), and the b* value indicates color changes from yellow (positive values) to blue (negative values) [44]. The results showed that the color parameters were significantly affected by the different formulations (*p* < 0.05). All color parameter (L*, a*, and b*) values were increased by increasing the percentage of the JWE powder. In the other words, the JWE powder enhanced the surface glossiness of GCs and made them more attractive to consumers. In addition, the they became more yellowish and less greenish. These results are in accordance with Romo-Zamarrón et al.’s [20] findings for the enrichment of the GCs with papaya powders.

In contrast, Altınok et al. [14] reported that the soft candies became darker with the addition of the grape seed and skin powder. In addition, the color parameters of GCs significantly affected by different concentrations of citric acid and agar gum but did not show a regular trend (*p* < 0.05). Previously, Hasani and Yazdanpanah [45] represented that the Cordia gum decreased the L* value of apple jelly and increased the b* value of samples but had no effect on the a* value. These changes may be due to the presence of some pigments in the gums that affected the color parameters of the final product.

#### 3.2.4. Viscosity

The rheology analysis affect the texture and sensory properties of the food product and is very important to optimizing the making processes, packaging, and storage tactics [14]. Viscosity is a property of the liquid materials and reflects their resistance to flow when exposed to a shear force. The thicker the liquid layer, the greater its resistance. The viscosity of liquids is also affected by temperature. Higher temperatures make it easier for fluids to flow, while lower temperatures cause an increase in viscosity. That is why it is important to measure the viscosity of a liquid at a constant temperature, and that temperature is specific to that material [46]. According to Table 4, JWE powder, citric acid, and agar gum can significantly (*p* < 0.05) influence the viscosity of GCs. In general, the viscosity of GCs increased by increasing the percentage of JWE powder and agar gum. The lowest viscosity was related to the GC 1 with 20% JWE powder, 0.75% citric acid, and 0.5% agar gum (0.457 Pa·s), while the highest value was related to the GC 10 with 50% JWE powder, 0.75% citric acid, and 1% agar gum (1.550 Pa·s). Altınok et al. [14] reported that the elasticity of soft candies was increased by adding the grape seed and skin, which strengthens the internal structure of the candies. Moreover, Yu et al. [47] demonstrated that the apparent viscosity of agar solution was pH-dependent and increased by increasing the pH value in the acidic area, which is our experimental range (See Table 3). In addition, the apparent viscosity of agar increased linearly with the increase in concentration between 0.5 and 1% because of the macromolecular aggregates and colloidal particles entanglement. These phenomena are correlated with our findings.

#### 3.2.5. Textural Properties

Texture properties are one of the most important quality indicators for confectionery products, which have crucial effects on consumer attitude and perception [14]. Moreover, the textural parameters of foodstuffs may be differ significantly at both microscopic and macroscopic scales due to the different interactions between the structural elements and the other components, as well as the varied processes [1,43]. In this study, the hardness (1667–7232 g), springiness (3.690–5.355 mm), cohesiveness (0.415–0.730), gumminess (928.3–3023.5 g), and chewiness (43.20–124.20 gmm) values of GCs containing different percentages of JWE powder, citric acid, and agar gum were evaluated (Table 5). As the results showed, the hardness, gumminess, and chewiness values of GCs significantly increased with increasing the percentage of JWE powder, citric acid, and agar gum (*p* < 0.05); the lowest values of these parameters were related to the GC 1 sample (20% JWE powder, 0.75% citric acid, and 0.5% agar gum) and the highest were related to the GC 12 sample (50% JWE powder, 1% citric acid, and 1% agar gum). However, the springiness and cohesiveness parameters did not show a regular trend.

Hardness is defined as a maximum force applied in the first compression to deform the sample [43]. Therefore, higher energy is required for the deformation of GCs by increasing the JWE powder, acetic acid, and agar gum concentrations. The thickening effect of JWE powder is consistent with the viscosity determination (See Table 4), which can be useful for its application as a thickening or texturing agent in jelly products made without other gelling agents [42]. Previously, Cappa et al. [8] showed that grape skin powder could modify the micro- and macronetwork of fruit candies and increase the required penetration energy. They suggested that the fiber organization and the pectin networking might be the reason for that phenomenon. In addition, Moghaddas Kia et al. [43] represented that the hardness of GCs increased with increasing the red beet extract content, which was accelerated by lowering the pH value. This may be due to the presence of phenolic compounds, sugar, and dry matters in the mixture. In addition, water can act as a plasticizer and form hydrogen bonds with other components such as agar gum in the gummy confections and soften the texture of the final product [48]. In the present study, the hardness value and water content (See Table 3) of GCs showed a negative correlation. In the other words, the hardness of GCs increased by decreasing the water content of the mixture. The same results were reported by Cappa et al. [8], previously. Moreover, Gok et al. [1] demonstrated that the crystallization is inhibited in the low free-water system, which increases the hardness.

Springiness is another important texture parameter, which shows the required mastication energy in the mouth [14]. GC 1 (20% JWE powder, 0.75% citric acid, and 0.5% agar gum) showed the highest springiness value, and GC 4 (35% JWE powder, 0.75% acetic acid, and 0.5% agar gum) represented the lowest. In addition, cohesiveness is required energy to tolerate food against deformation. By increasing the percentages of all treatments, the cohesiveness was increased except by increasing the JWE powder from 35% to 50%. Previously, Altınok et al. [14] reported that grape-based wastes did not affect the cohesiveness parameter significantly. Gumminess (the energy needed for the disintegration of semisolid foods) is evaluated by multiplying hardness and cohesiveness [43]. In addition, chewiness (the required energy to make the solid food ready for swallowing) is determined by multiplying hardness, cohesiveness, and springiness [14]. Therefore, chewiness values were more important than gumminess values in GC products [42]. Moreover, the hardness value plays a key role in the evaluation of gumminess and chewiness values. As mentioned, the hardness, gumminess, and chewiness have the same trend in the present study. The increase in these values may be due to the homogenous structure of JWE powder that makes the final network more ordered [43]. In addition, da Costa et al. [49] reported that the fruit pulp makes the agar gels less firm and brittle, which had no correlation with our results, and the JWE powder and agar gum showed a synergistic pattern. These results represent that the JWE powder, citric acid, and agar gum can affect the texture parameters of GCs in different ways and generally make the final product firmer and more elastic.

#### 3.2.6. Sensory Properties of GCs

**Figure 1:**
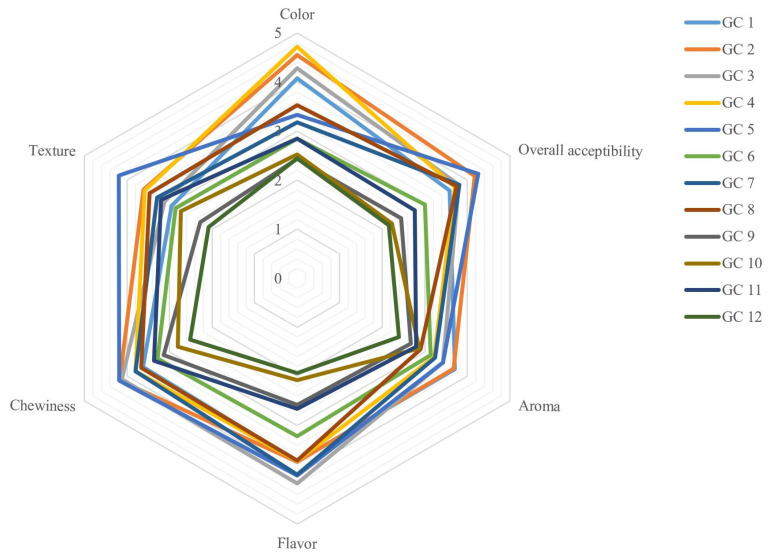
Sensory properties of different GCs.

The confectionery products are generally consumed for pleasure. Moreover, the consumer’s demand increased for functional and healthier products, which are enriched with bioactive and fiber components [1]. Therefore, the sensory characteristics of high-fiber GC are important for product development as a delicious and nutritious product. The sensory properties (color, texture, chewiness, flavor, aroma, and overall acceptability) of GCs with different concentrations of JWE powder, citric acid, and agar gum are represented in Figure 1. Unlike citric acid, all sensory parameters of GCs were significantly affected by different percentages of JWE powder and agar gum (*p* < 0.05). The color acceptability of GCs generally decreased by increasing the JWE powder and agar gum contents. The lowest scores for color acceptability of GCs were determined for the GC 9, 10, and 12 samples and the highest value was evaluated for the GC 5 sample. These findings are not totally compatible with the color properties that were evaluated by instrumental measurements. According to our results, the panelists scored almost similar to the texture and chewiness parameters. Moreover, agar gum enhanced the texture and chewiness values in all doses, but it showed an antagonism effect with fiber at 50% of JWE powder, which makes the GCs firmer and requires more energy for swallowing. In addition to the fiber content of GCs, the low moisture content of these GCs can negatively affect the panelist’s pleasure [7]. The aroma and flavor of GCs are the other important sensory properties. JWE powder and agar gum significantly reduced these parameters (*p* < 0.05). These results are in correlation with Gok et al.’s [1] findings. They represented that less flavor was released from harder GCs, which have a higher amount of fiber. In addition, Lubbers and Guichard [50] demonstrated that water activity and viscosity are two important parameters in GCs, which can affect the release of aroma and flavor compounds. Although hydrocolloids such as agar gum can modify the viscosity and textural properties of products, they can also reduce the intensity of the sample’s aroma and flavor by acting as a barrier and inhabiting the aroma and flavor component diffusion, and this affects overall acceptance. The lowest overall acceptability scores were given to GC 10 and GC 12 samples (2.23 ± 0.5 and 2.15 ± 0.31, respectively), and the highest score was related to GC 5 sample (4.26 ± 0.01). According to these results, the panelists generally preferred softer GCs with medium percentages of JWE powder and low citric acid and agar gum contents. Similarly, these results were obtained by Romo-Zamarrón et al. [20], which reported that the panelists preferred the softer GCs than fiber-rich candies.

## 4. Conclusions

The JWE powder is rich in dietary fiber, minerals, phenolic components, and vitamin C, which makes it a promising source for application in functional products. The current research successfully demonstrated that the JWE powder, in combination with citric acid and agar gum, can potentially be used in the GC production to meet consumer demand for healthier confectionery products. By increasing the percentages of JWE powder in GC formulation, the moisture content and water activity of the GCs decreased, while the pH value increased, which enhances the shelf life of the final product. In addition, the surface of GCs became brighter and more attractive. The viscosity and textural properties of enriched GCs showed that the JWE powder can act as a texturizing agent and increase the firmness of GCs. Nevertheless, the sensory evaluation of different GCs represented that the panelists preferred softer GCs, with medium percentages of JWE powder and low citric acid and agar gum contents.

## Figures and Tables

**Table 1:** The formulation of different GCs.

Samples	JWE Powder	Citric Acid	Agar Gum	Pectin	Arabic Gum	Sucrose	Glucose	Starch	Water
GC 1	20	0.75	0.5	0.541	0.541	20	10	0.18	47.488
GC 2	20	0.75	1	0.541	0.541	20	10	0.18	46.988
GC 3	20	1	0.5	0.541	0.541	20	10	0.18	47.238
GC 4	20	1	1	0.541	0.541	20	10	0.18	46.738
GC 5	35	0.75	0.5	0.541	0.541	20	10	0.18	32.488
GC 6	35	0.75	1	0.541	0.541	20	10	0.18	31.988
GC 7	35	1	0.5	0.541	0.541	20	10	0.18	32.238
GC 8	35	1	1	0.541	0.541	20	10	0.18	31.738
GC 9	50	0.75	0.5	0.541	0.541	20	10	0.18	17.488
GC 10	50	0.75	1	0.541	0.541	20	10	0.18	16.988
GC 11	50	1	0.5	0.541	0.541	20	10	0.18	17.238
GC 12	50	1	1	0.541	0.541	20	10	0.18	16.738

**Table 2:** Chemical composition and antioxidant activity of JWE powder.

Value	Measurement Index
Vitamin C (mg/100 g)	55.19 ± 0.41
TPC (mg gallic acid/g)	52.62 ± 0.34
TAC (mg cyanidin-3-glucoside/100 g)	2.56 ± 0.03
TFC (mg catechin/100 g)	36.21 ± 0.12
TAA (%)	25.06 ± 0.27

TPC: total phenolic content; TAC: total anthocyanin content; TFC: total flavonoid content; TAA: total antioxidant activity.

**Table 3:** Moisture content, water activity, and pH values of different GCs.

Samples	Moisture Content (g/100 g)	Water Activity	pH
GC 1	29.408 ± 0.354 ^a^	0.815 ± 0.006 ^a^	2.735 ± 0.078 ^bcd^
GC 2	27.586 ± 1.087 ^a^	0.792 ± 0.004 ^ab^	2.725 ± 0.035 ^bcd^
GC 3	27.820 ± 1.393 ^a^	0.777 ± 0.001 ^b^	2.415 ± 0.064 ^de^
GC 4	28.580 ± 1.563 ^a^	0.793 ± 0.002 ^ab^	2.370 ± 0.085 ^e^
GC 5	26.988 ± 0.178 ^abc^	0.763 ± 0.017 ^bc^	2.950 ± 0.014 ^abc^
GC 6	26.607 ± 0.562 ^abc^	0.771 ± 0.001 ^b^	2.975 ± 0.007 ^abc^
GC 7	27.457 ± 0.609 ^ab^	0.790 ± 0.011 ^ab^	2.680 ± 0.226 ^cde^
GC 8	26.745 ± 0.147 ^abc^	0.774 ± 0.008 ^b^	2.790 ± 0.014 ^bc^
GC 9	24.347 ± 0.245 ^cd^	0.732 ± 0.003 ^cd^	3.205 ± 0.071 ^a^
GC 10	24.120 ± 0.035 ^cd^	0.768 ± 0.001 ^b^	3.150 ± 0.071 ^a^
GC 11	24.613 ± 0.143 ^bcd^	0.725 ± 0.020 ^de^	2.985 ± 0.007 ^abc^
GC 12	23.348 ± 0.470 ^d^	0.695 ± 0.010 ^e^	3.025 ± 0.035 ^ab^

GC: gummy candy. In each column, means with same letters had no significant difference with each other (*p* > 0.05).

**Table 4:** The color characteristics and viscosity of different GCs.

Samples	L*	a*	b*	Viscosity (Pa·s)
GC 1	70.86 ± 4.83 ^d^	−12.81 ± 0.25 ^c^	14.99 ± 0.20 ^ef^	0.457 ± 0.002 ^k^
GC 2	70.71 ± 2.77 ^d^	−12.39 ± 0.04 ^c^	15.36 ± 0.98 ^e^	0.687 ± 0.005 ^i^
GC 3	70.87 ± 5.65 ^d^	−12.09 ± 0.54 ^c^	13.43 ± 0.75 ^f^	0.810 ± 0.003 ^g^
GC 4	72.05 ± 2.35 ^d^	−12.40 ± 0.45 ^c^	14.36 ± 0.03 ^f^	0.795 ± 0.005 ^h^
GC 5	83.18 ± 3.01 ^a^	−10.22 ± 0.34 ^ab^	14.43 ± 0.29 ^f^	1.183 ± 0.003 ^c^
GC 6	79.14 ± 4.40 ^b^	−10.67 ± 0.85 ^ab^	18.43 ± 0.99 ^d^	0.848 ± 0.003 ^f^
GC 7	82.62 ± 6.77 ^a^	−11.03 ± 0.75 ^b^	17.90 ± 0.33 ^d^	0.543 ± 0.006 ^j^
GC 8	79.81 ± 3.87 ^ab^	−10.91 ± 0.26 ^ab^	19.52 ± 0.19 ^c^	1.018 ± 0.005 ^d^
GC 9	82.56 ± 1.09 ^a^	−10.15 ± 0.46 ^ab^	18.34 ± 0.46 ^d^	1.385 ± 0.008 ^b^
GC 10	77.30 ± 2.86 ^bc^	−10.27 ± 0.13 ^ab^	24.24 ± 0.40 ^a^	1.550 ± 0.008 ^a^
GC 11	75.48 ± 4.09 ^c^	−10.66 ± 0.92 ^ab^	24.55 ± 0.54 ^a^	0.940 ± 0.003 ^e^
GC 12	78.43 ± 5.91 ^bc^	−9.97 ± 0.52 ^a^	21.70 ± 0.64 ^b^	1.184 ± 0.003 ^c^

GC: gummy candy. In each column, means with same letters had no significant difference with each other (*p* > 0.05).

**Table 5:** Textural properties of different GCs.

Samples	Hardness (g)	Springiness (mm)	Cohesiveness	Gumminess (g)	Chewiness (gmm)
GC 1	1667 ± 120 ^d^	5.355 ± 0.021 ^a^	0.420 ± 0.003 ^e^	928.3 ± 157 ^e^	43.20 ± 1.21 ^e^
GC 2	2101 ± 230 ^d^	4.085 ± 0.076 ^cde^	0.615 ± 0.007 ^bc^	1287.0 ± 345 ^de^	58.95 ± 1.87 ^de^
GC 3	2253 ± 654 ^d^	3.875 ± 0.065 ^de^	0.670 ± 0.003 ^ab^	1283.5 ± 760 ^de^	46.65 ± 1.01 ^e^
GC 4	3595 ± 435 ^bcd^	3.690 ± 0.023 ^e^	0.695 ± 0.001 ^ab^	2498.5 ± 125 ^ab^	72.70 ± 2.08 ^d^
GC 5	3934 ± 128 ^bcd^	4.455 ± 0.012 ^bcd^	0.625 ± 0.010 ^b^	2451.0 ± 659 ^abc^	94.55 ± 1.02 ^c^
GC 6	2446 ± 107 ^cd^	4.025 ± 0.010 ^de^	0.730 ± 0.011 ^a^	1775.5 ± 234 ^bcde^	67.35 ± 6.20 ^d^
GC 7	2164 ± 802 ^d^	4.395 ± 0.062 ^bcd^	0.615 ± 0.009 ^bc^	1308.5 ± 222 ^cde^	56.45 ± 3.65 ^de^
GC 8	4572 ± 700 ^bc^	4.830 ± 0.012 ^ab^	0.540 ± 0.005 ^cd^	2280.5 ± 896 ^abcd^	115.25 ± 2.51 ^ab^
GC 9	4686 ± 203 ^bc^	4.790 ± 0.043 ^ab^	0.435 ± 0.001 ^e^	2232.0 ± 450 ^abcd^	103.60 ± 1.90 ^bc^
GC 10	5779 ± 513 ^ab^	4.720 ± 0.019 ^abc^	0.470 ± 0.003 ^de^	2708.0 ± 65 ^ab^	125.35 ± 1.33 ^a^
GC 11	4835 ± 129 ^b^	4.830 ± 0.011 ^ab^	0.475 ± 0.006 ^de^	2290.0 ± 102 ^abcd^	116.80 ± 2.23 ^ab^
GC 12	7232 ± 345 ^a^	4.475 ± 0.029 ^bcd^	0.415 ± 0.002 ^e^	3023.5 ± 603 ^a^	124.20 ± 1.13 ^a^

GC: gummy candy. In each column, means with same letters had no significant difference with each other (*p* > 0.05).

## Data Availability

The data presented in this study are available upon request from the corresponding author.
